# Transcriptomic Identification of Key Genes Responding to High Heat Stress in Moso Bamboo (*Phyllostachys edulis*)

**DOI:** 10.3390/genes16080855

**Published:** 2025-07-23

**Authors:** Qinchao Fu, Xinlan Wen, Man Tang, Xin Zhao, Fang Liu

**Affiliations:** 1 Natural Sciences Museum of Leshan Normal University, Key Laboratory of Sichuan Province for Bamboo Pests Control and Resource Development, Leshan Normal University, Leshan 614000, China; fuqinchao@lsnu.edu.cn (Q.F.); 18090377485@163.com (X.W.); 2 College of Life Science and Agri-Forestry, Southwest University of Science and Technology, Mianyang 621000, China; 18224205087@163.com; 3 Sichuan Provincial Forestry and Grass Land Key Laboratory for Conservation and Sustainable Utilization of Bamboo Genetic Resources in Southwest of China, Mianyang 621000, China; 4 School of Life Science, Leshan Normal University, Leshan 614000, China

**Keywords:** Moso bamboo (*Phyllostachys edulis*), heat stress responses, key genes, regulatory network

## Abstract

**Background/Objectives:** Moso bamboo (*Phyllostachys edulis*), the most widely distributed bamboo species in China, is valued for both its shoots and timber. This species often faces challenges from high-temperature stress. To cope with this stress, Moso bamboo has evolved various adaptive mechanisms at the physiological and molecular levels. Although numerous studies have revealed that a large number of transcription factors (TFs) and genes play important roles in the regulatory network of plant heat stress responses, the regulatory network involved in heat responses remains incompletely understood. **Methods:** In this study, Moso bamboo was placed in a high-temperature environment of 42 °C for 1 h and 24 h, and transcriptome sequencing was carried out to accurately identify key molecules affected by high temperature and their related biological pathways. **Results:** Through a differential expression analysis, we successfully identified a series of key candidate genes and transcription factors involved in heat stress responses, including members of the ethylene response factor, HSF, WRKY, MYB, and bHLH families. Notably, in addition to traditional heat shock proteins/factors, multiple genes related to lipid metabolism, antioxidant enzymes, dehydration responses, and hormone signal transduction were found to play significant roles in heat stress responses. To further verify the changes in the expression of these genes, we used qRT-PCR technology for detection, and the results strongly supported their key roles in cellular physiological processes and heat stress responses. **Conclusions:** This study not only deepens our understanding of plant strategies for coping with and defending against extreme abiotic stresses but also provides valuable insights for future research on heat tolerance in Moso bamboo and other plants.

## 1. Introduction

Global warming has become one of the most serious challenges facing the world today, with far-reaching impacts on ecosystems and agricultural production [1]. As temperatures continue to rise, extreme heat events are becoming increasingly frequent, leading to the deterioration of the plant growth environment and the serious disruption of physiological and metabolic processes [2,3,4]. High-temperature stress not only leads to decreased photosynthetic efficiency and increased respiration but also affects water absorption and utilization and the stability of the membrane system, which in turn has a serious impact on plant growth, development, and yield [5]. Therefore, an in-depth understanding of how plants respond and adapt to heat stress, and the revelation of the underlying molecular mechanisms are of great significance for improving heat tolerance in plants and ensuring food security and ecological balance [6,7].

As one of the most ecologically and economically valuable bamboo species in China, Moso bamboo (*Phyllostachys edulis*) is strategically important in addressing global warming because of its fast growth and carbon sink function [8,9,10,11]. However, the frequent occurrence of extreme heat events in recent years has had several adverse effects on Moso bamboo, including a decline in photosynthetic efficiency, metabolic disorders, and even death, seriously threatening its ecological function and industrial development [12]. Over the long course of evolution, this plant species has developed a set of complex and efficient strategies to cope with this adversity so as to maintain normal physiological, metabolic, and growth functions [13,14]. At the transcriptional level, the regulation of gene expression and the rapid control of metabolites play important roles in plant stress resistance [15,16]. For example, heat shock transcription factors (HSFs), a class of transcription factors ubiquitous in eukaryotes, not only play important roles in the perception and transmission of signals, the recognition of heat shock elements (HSEs) [17], and the regulation of downstream genes but also participate in the regulation of reactive oxygen species (ROS), calcium signals, and other related signaling pathways [18]. Notably, they are of great significance in triggering plant stress responses and enhancing heat tolerance. ABRE binding factors (ABFs) and MYC transcription factors play key roles in the abscisic acid (ABA) and jasmonic acid (JA) signaling pathways, respectively [16,19,20]. Next, when plants respond to high-temperature stress, their molecular mechanisms mainly rely on the involvement of heat shock proteins (HSPs), which play a key role in plant adaptation and survival. The tolerance of plants to high temperatures and their adaptation to continuous heat stress depend on whether heat shock proteins can be expressed in a timely manner and accumulate effectively [21,22].

Although studies have focused on plant response mechanisms to heat stress, most of the research has been primarily focused on the regulation of core transcription factors such as HSFs, and there may be other key genes that have been overlooked [10,23,24,25,26]. The specific molecular mechanisms of the response to heat stress in Moso bamboo need to be further explored. In particular, how Moso bamboo regulates gene expression to respond and adapt to heat stress, as well as which key genes play important roles in this process, are issues that urgently need to be resolved. Therefore, the present study aims to reveal the molecular response mechanism of Moso bamboo under heat stress and provide a scientific basis for genetic improvement and cultivation management of Moso bamboo.

In this study, we integrated transcriptome sequencing, differential gene expression analysis, and functional enrichment to systematically screen for genes that play a key role in the response to heat stress in Moso bamboo. By comparing leaf samples stressed at 42 °C for 0 h (control), 1 h (acute stress), and 24 h (continuous stress), we revealed temporal changes in gene regulation, including the activation of heat stress proteins (HSPs) and transcription factors (e.g., HSF, MYB, and NAC families), as well as stress-associated pathways, such as the MAPK signaling and phenylpropane biosynthesis pathways. The key candidate genes were further validated using real-time quantitative PCR (qRT-PCR) to confirm their important roles in heat acclimation. This study not only deepens the understanding of the molecular mechanism of heat stress in Moso bamboo but also provides targets for breeding stress-resistant varieties to aid in its sustainable utilization in a warming climate.

## 2. Materials and Methods

### 2.1. Plant Materials and Heat Treatment Methods

Seedlings of *Phyllostachys edulis* were planted in the greenhouse of Southwest University of Science and Technology. Four-month-old Phyllostachys edulis seedlings were subjected to heat treatment at 42 °C for 1 h or 24 h, with the experimental groups labeled as CK, T1, and T2, corresponding to the control group, 1-h treatment, and 24-h treatment, respectively. The *Phyllostachys edulis* seedlings were grown in groups of three, with a total of three groups, under cultivation conditions of 400 μmol/m^2^/s light intensity, humidity of 60–70%, soil water content of 60–70%, and a 16-h light/8-h dark photoperiod. The control group was grown at 25 °C.

### 2.2. RNA Extraction and Transcriptome Sequencing

Total RNA was extracted using the plant RNA extraction kit (R6827, Omega Bio-Tek, Norcross, GA, USA). RNA quality was evaluated using a NanoDrop One Spectrophotometer (Thermo Fisher Scientific, Wilmington, NC, USA) to assess OD260/280 and OD260/230 ratios, followed by quantification with a Qubit 3.0 Fluorometer (Life Technologies, Carlsbad, CA, USA). Only RNA samples with an Concentration ≥ 20 ng/μL, total RNA > 1 μg, RQN > 4.5 OR Concentration ≥ 10 ng/μL, total > 10 ng, RIN > 6.5, were utilized for subsequent cDNA library preparation using the MGIEasy RNA Library Preparation Kit (MGI Tech Co., Ltd., Shenzhen; China). During cDNA library preparation, mRNA was first enriched using oligo(dT) magnetic beads, followed by fragmentation with a fragmentation reagent to generate mRNA fragments. Using the fragmented mRNAs as templates, first-strand cDNA was synthesized with reverse transcriptase, followed by second-strand synthesis via PCR. The double-stranded cDNA was then processed through end repair (using T4 DNA polymerase), 3’-A-tailing (with Klenow fragment), adapter ligation, and subsequent purification/amplification steps to complete library construction. The constructed libraries were analyzed for fragment size and concentration using an Agilent 2100 Bioanalyzer (Agilent Technologies, Santa Clara, CA, USA). Qualified libraries were sequenced on DNBSEQ-T7 platform(PE150) using DNBSEQ-T7RS Reagent Kit (FCL PE150) version 3.0. The raw sequencing data supporting the findings of this study have been deposited in the NCBI Sequence Read Archive (SRA) under the BioProject accession number PRJNA1289125. These data are publicly available and can be accessed through the NCBI SRA database.

### 2.3. Transcriptome Assembly and Unigene Annotation

To ensure the accuracy and reliability of the analysis, the raw sequencing data were subjected to rigorous quality control and meticulous preprocessing procedures. This involved filtering out low-quality reads, specifically those containing more than five N bases, reads where the number of low-quality bases accounted for 50% or more of the read length, reads contaminated with adapter sequences, and repetitive sequences resulting from PCR amplification. Subsequently, quality control of the filtered data was performed using FastQC (version 0.11.9) with default parameters to ensure the data met the standards required for subsequent analysis (e.g., Phred quality score ≥ 30, sequence length ≥ 50 bp, and adapter contamination < 1%). The clean reads obtained from the quality control step were then utilized for transcript assembly using Trinity (version 2.11.0, with the parameter −min_kmer_cov 2 set). Following this, the assembled transcripts were clustered using cd-hit (version v4.8.1, with parameters −c 0.85 and −aS 0.85) to generate non-redundant genes, or unigenes. To evaluate the completeness of unigenes, BUSCO analysis was first performed to assess gene set integrity. Furthermore, the coding regions of the unigenes were predicted using TransDecoder software (version v5.7.1). For a comprehensive functional annotation of the unigenes, seven databases were utilized for motif and sequence similarity searches. These databases included GO, Nr, KOG/COG, Pfam, Uniprot, KEGG, and PATHWAY. Additionally, transcription factors were identified using the plant-specific database PlantTFDB (version 5.0).

### 2.4. Measurement of Chlorophyll Fluorescence Parameters

The determination of maximal quantum yield of PSll photochemistry (Fv/Fm), effective quantum yield of PSII photochemistry (Y(ll)), photochemical quenching coefficient (qP) and non-photochemical quenching (NPQ) were measured with fluorometer MIN-PAM (Heinz Walz GmbH, Effeltrich, Germany) according to the instructions. After 30 min of dark adaptation, the initial fluorescence (Fo), maximum fluorescence yield (Fm), minimum light-adapted fluorescence yield (Fo′) and maximum light-adapted fluorescence yield (Fm′) values were recorded. According to the calculation of Fv/Fm = (Fm − Fo)/Fm, Yll = (Fm′ − Fo)/Fm′, NPQ = (Fm − Fm′)/Fm′, qP = (Fm − Fm′)/(Fm − Fo).

### 2.5. Quantitative Real-Time PCR (qRT-PCR) Validation

To validate the gene expression obtained from high-throughput sequencing, specific primers were designed using Primer Premier 5.0 software based on the candidate gene sequences (Table S1). RT-qPCR reactions were performed on a qTOWER 2.2 system (Analytik Jena, Jena, Germany) using SYBR Green Master Mix (Vazyme Biotech Co., Ltd., Nanjing, China), with three technical replicates for each sample. Actin was used as the internal reference gene. The PCR conditions were as follows: 95 °C for 10 min, followed by 40 cycles of 95 °C for 10 s and 62 °C for 10 s. The 2^−^ᴰᴰᶜᵀ method was used to calculate the expression levels.

### 2.6. Statistical Analysis

Statistical analyses were performed using GraphPad Prism 10 (GraphPad Software, San Diego, CA, USA). A one-way analysis of variance (ANOVA) followed by post hoc tests (e.g., Tukey’s or Dunnett’s test) was used to assess variance and significant differences among groups. The data are presented as the mean ± standard deviation (SD) of three biological replicates. Statistical significance was set at *p* < 0.05.

## 3. Results

### 3.1. Detection of Photosynthetic Rate After High-Temperature Stress

**Figure 1:**
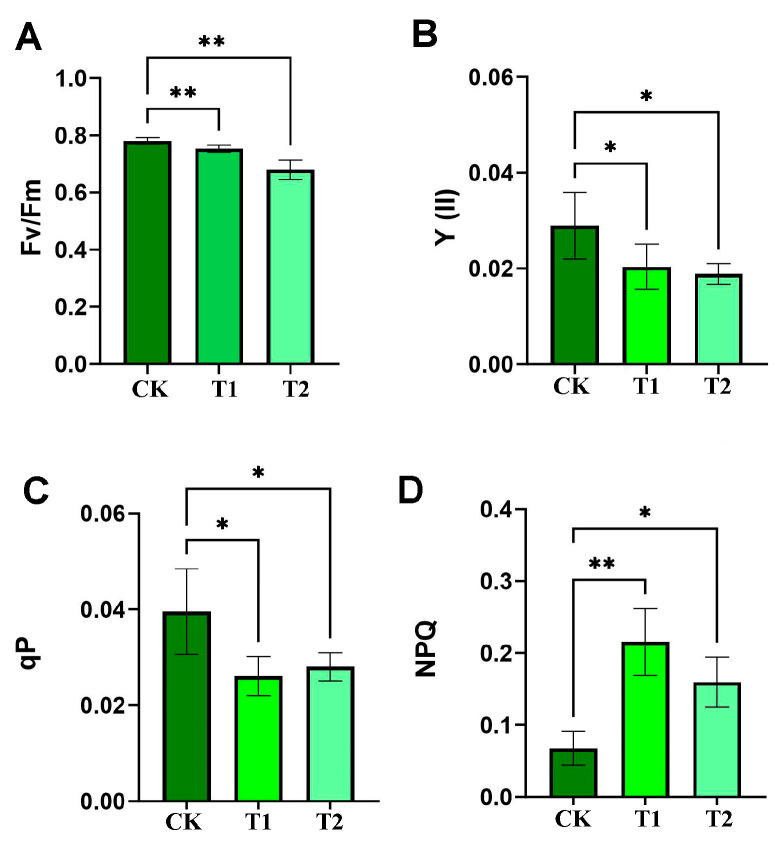
Detection of chlorophyll fluorescence indexes after high-temperature stress in Moso bamboo leaves. (**A**) Fv/Fm, maximum quantum yield of PSII photochemistry. (**B**) Y(II), effective quantum yield of PSII photochemistry. (**C**) qP, photochemical quenching coefficient. (**D**) NPQ, non-photochemical quenching. Error bars indicate averages of measurements from at least three biological replicates, and asterisks indicate significant differences. Significant differences were analyzed using the one-way ANOVA method, * *p* < 0.05, ** *p* < 0.01. CK, 25 °C; T1, 42 °C for 1 h; T2, 42 °C for 24 h, same for subsequent figures unless noted. Error bars indicate averages of measurements from at least three biological replicates, and asterisks indicate significant differences. Significant differences were analyzed using the one-way ANOVA method, * *p* < 0.05, ** *p* < 0.01.

High-temperature stress typically results in the attenuation of metabolic processes in plants, including photosynthesis, respiration, and water absorption and utilization. Chlorophyll fluorescence is a key technique in plant photosynthesis research, and its measurements can non-destructively reflect the energy conversion efficiency of the photosynthetic system in real time, with a strong physiological correlation with photosynthetic rate, especially the rate of CO_2_ fixation. In this study, the values of Fv/Fm, Y (II), and qP exhibited a notable decline under high-temperature conditions (Figure 1A–C), while the value of NPQ increased significantly (Figure 1D). These findings suggest that high-temperature stress markedly impaired the photosynthetic capacity of the Moso bamboo, with the inhibitory effect on photosynthesis commencing within 1 h and rapidly reaching its peak.

### 3.2. Transcriptome Sequencing and Gene Functional Characterization

To further elucidate the molecular mechanisms underlying the rapid response of Moso bamboo to high-temperature stress, we collected leaf samples from Moso bamboo seedlings subjected to high-temperature stress at 42 °C for 0 h (control, CK), 1 h (T1), and 24 h (T2). Subsequently, we sequenced the transcriptomes of these samples.

In this study, transcriptome sequencing was successfully completed for nine samples, yielding a total of 67.71 Gb of Clean Data. Each sample generated more than 6.38 Gb of Clean Data, with a Q30 base percentage exceeding 96.84%. The Q20 rate, indicating the proportion of bases with a Phred value of at least 20, was above 99.04%. Furthermore, the GC content of the samples ranged from 48.2% to 50.9% (Table S2). Separate sequence alignment was performed for the clean reads of each sample against the designated reference genome, resulting in alignment rates ranging from 93.8% to 94.98% (Table S3).

### 3.3. Unigene Annotation and Transcription Factor (TF) Identification

**Figure 2:**
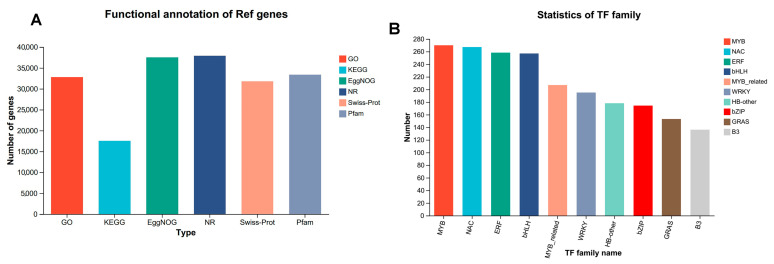
Gene annotation and transcription factor identification statistics. (**A**) Gene annotation statistics for 6 major databases. (**B**) Statistics on the number of major transcription factor families.

In this analysis, a total of 47,035 expressed genes were identified, comprising 38,673 known genes and 8362 novel genes. Additionally, 80,641 expressed transcripts were detected, including 37,172 known transcripts and 43,469 novel transcripts. Among the 38,673 expressed genes identified in the transcriptome data, 37,964 genes were successfully annotated across seven major databases. Specifically, 37,504 genes were annotated in the EggNOG database, accounting for 96.98% of the annotated genes. The number of successfully annotated genes in the remaining databases were as follows: GO (32,759, 84.71%), KEGG (17,547, 45.37%), NR (37,905, 98.01%), Swiss-Prot (31,795, 82.21%), and Pfam (33,388, 86.33%) (Table S4 and Figure 2A).

Furthermore, utilizing the Plant Transcription Factor Database Comparison, a total of 3269 transcription factors were identified. The highest number of transcription factors belonged to the MYB family, with 270 members, followed by the NAC family (267), the bHLH family (257, also highly represented), and the ERF family (258, which also constitutes a significant number of transcription factors). In contrast, transcription factors in the AP2 and GRF families were less represented, with only 46 in the AP2 family and 23 in the GRF family. Overall, there were approximately 50 transcription factors in the ARF and TCP families, while there were 32 in HD-ZIP family, 58 in the HSF family, 195 in the WRKY family, 174 in the bZIP family, and 153 in the GRAS family, reflecting the diverse distribution of transcription factor numbers across different families in the organism. These variations may be closely associated with their roles in distinct physiological functions, such as growth and development and environmental responses. Families with a higher number of transcription factors may be involved in more complex and extensive regulatory tasks within the relevant regulatory processes (Table S5 and Figure 2B).

### 3.4. Analysis of Differentially Expressed Genes (DEGs)

**Figure 3:**
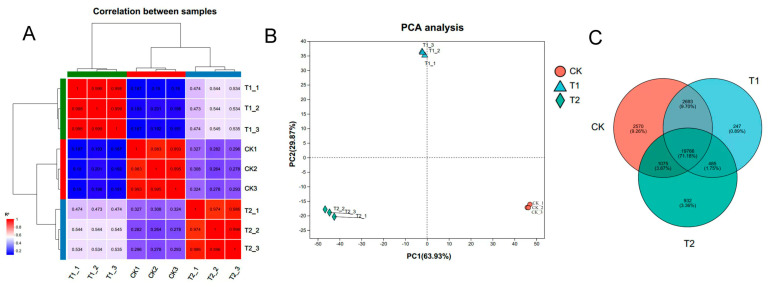
Correlation analysis between transcriptome samples. (**A**) A heatmap of correlations between samples. The right and bottom sides of the figure show the names of the samples, the left and top sides show the clustering of the samples, and differently colored squares represent the high or low correlation between the two samples. (**B**) Principal component analysis (PCA) of the samples. The distance of each sample point represents the distance between samples, with closer distances indicating higher similarity between them. (**C**) Venn analysis of the samples. Venn analysis demonstrates the number of genes/transcripts in each group of samples and the overlap of genes/transcripts between groups of samples; the distribution of the number of genes/transcripts in each group of samples can be visualized using Venn analysis.

To assess the quality and usability of the transcriptome data, we examined the correlation among individual samples within the dataset, as depicted in Figure 3. The heatmap illustrating sample correlations reveals that the correlation coefficient between biological replicates exceeded 97%, while notable differences were observed between samples from distinct treatment groups (Figure 3A). Furthermore, the results of the principal component analysis (PCA) demonstrated that biological replicates were clearly grouped within the same cluster (Figure 3B). A Venn diagram analysis of expressed genes across the three treatment samples (CK, T1, and T2) indicated that 19,766 genes were commonly expressed in all three groups. In contrast, 2570, 247, and 932 genes were uniquely expressed in the CK, T1, and T2 samples, respectively, potentially serving as key genes in the heat stress response (Figure 3C).

### 3.5. Analysis of Differentially Expressed Genes (DEGs) Among Transcriptomes

**Figure 4:**
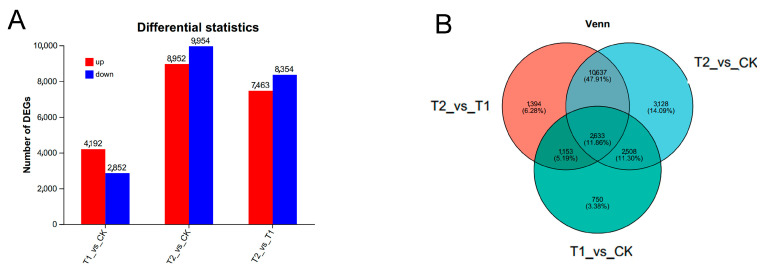
Analysis of differentially expressed genes among transcriptomes. (**A**) Differential expression statistics of samples from each transcriptome. The horizontal axis represents the different differential comparison groups, and the vertical axis represents the corresponding number of up- and downregulated genes/transcripts. Red represents upregulation and blue represents downregulation. (**B**) Venn analysis of differentially expressed genes among transcriptome samples. Differently colored circles represent different gene sets, and the values represent the number of genes/transcripts that are shared and unique between different gene sets.

To identify key genes involved in the heat stress response of Moso bamboo, we conducted a further analysis of differentially expressed genes (DEGs) among transcriptomes from the samples. As illustrated in Figure 4, the number of upregulated genes in the T1 vs. CK, T2 vs. CK, and T2 vs. T1 comparisons was 4192, 8952, and 7463, respectively, while the number of downregulated genes was 2852, 9954, and 8354, respectively (Figure 4A and Tables S6 and S7). Among the three groups of samples, 2633 DEGs were shared, whereas 750, 3128, and 1394 unique DEGs were identified in the T1 vs. CK, T2 vs. CK, and T2 vs. T1 groups, respectively (Figure 4B). These findings suggest that a limited number of genes responded promptly to the 1-h heat stress treatment and that an increasing number of genes were subsequently induced over time to coordinate the physiological response to heat stress.

**Figure 5:**
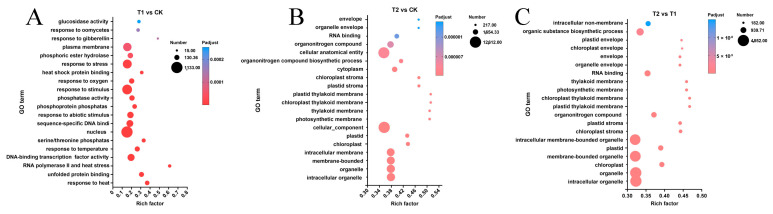
GO enrichment analysis of differentially expressed genes among transcriptomes. (**A**) List of the top 20 functional entries after GO enrichment analysis of DEGs in the T1 vs. CK group. (**B**) List of the top 20 functional entries after GO enrichment analysis of DEGs in the T2 vs. CK group. (**C**) List of the top 20 functional entries after GO enrichment analysis of DEGs in the T2 vs. T1 group.

**Figure 6:**
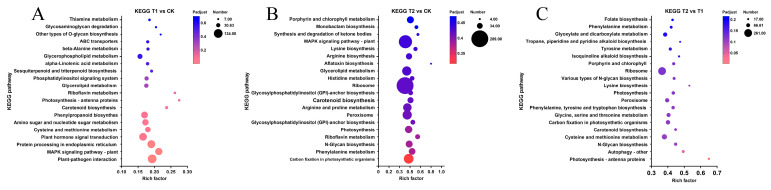
KEGG enrichment analysis of differentially expressed genes among transcriptomes. (**A**) List of the top 20 metabolic pathways after KEGG enrichment analysis of DEGs in the T1 vs. CK group. (**B**) List of the top 20 metabolic pathways after KEGG enrichment analysis of DEGs in the T2 vs. CK group. (**C**) List of the top 20 metabolic pathways after KEGG enrichment analysis of DEGs in the T2 vs. T1 group.

To identify key differentially expressed genes (DEGs) associated with critical metabolic pathways, we conducted gene enrichment analyses of GO and KEGG pathways for DEGs in each sample group. The top 20 metabolic pathways in each category, as revealed by the gene pathway enrichment analysis, are presented in Figure 5 and Figure 6. When plants were subjected to transient heat stress compared with the control (CK), the cellular component categories, including nucleus components, membranes, and membrane-intrinsic components, exhibited the highest number of altered single genes (Figure 5A). Upon exposure to 24 h heat stress, DEGs were further enriched in membranes such as chloroplasts, membrane-intrinsic components, and membrane-integrative components, in contrast to transient heat stress (Figure 5B). Notably, the primary disparities between prolonged heat stress and short-term heat stress were also evident in changes observed in plastid components and membrane components (Figure 5C).

The KEGG metabolic pathway enrichment analysis revealed the top 20 metabolic pathways in the comparison (Figure 6). Among these, the metabolic pathways with the most differentially expressed genes (DEGs) during the short-term heat stress response were plant–pathogen interaction, MAPK signaling pathway-plant, protein processing in plants, plant hormone signal transduction, and phenylpropanoid biosynthesis, all of which are closely associated with stress responses (Figure 6A). As the duration of heat stress increased, the predominant metabolic pathways shifted to ribosome, MAPK signaling pathway-plant, carbon fixation in photosynthetic organisms, peroxisome, and arginine and proline metabolism, which play crucial roles in further regulating intracellular homeostasis (Figure 6B). In contrast, the primary distinction between the responses to prolonged and short-term heat stress was evident in the ribosome metabolism pathway, which may suggest the onset of gradual apoptosis (Figure 6C).

### 3.6. Screening of Key Differentially Expressed Genes for Heat Stress

**Figure 7:**
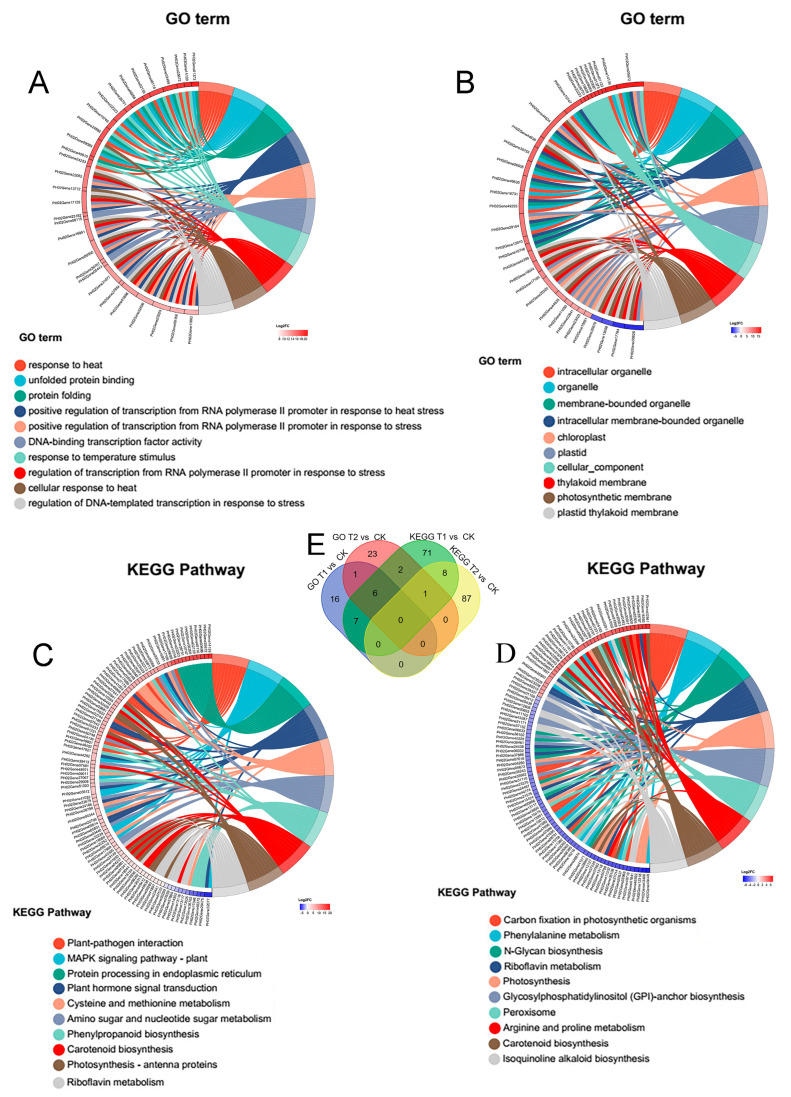
GO and KEGG gene set enrichment chordal maps with Venn analysis. (**A**) GO enrichment analysis and top genes screened in T1 vs. CK group. (**B**) GO enrichment analysis and top genes screened in T2 vs. CK group. (**C**) KEGG enrichment analysis and top genes screened in T1 vs. CK group. (**D**) KEGG enrichment analysis and top genes screened in T2 vs. CK group. (**E**) Venn analysis of gene sets after GO and KEGG enrichment analysis in T1 vs. CK and T2 vs. CK groups. Detailed information can be found in Tables S6 and S7.

To elucidate the Gene Ontology (GO) terms or pathways predominantly enriched by the target gene sets identified in this study and gain a deeper understanding of Moso bamboo’s response to heat stress, we employed transcriptome sequencing to qualitatively and quantitatively characterize the expression patterns of genes affected by heat stress in Moso bamboo. As illustrated in Figure 7, the differentially expressed gene sets in the T1 vs. CK and T2 vs. CK sample groups exhibited significant enrichment in GO and KEGG metabolic pathways. We selected the top 10 most significant components and metabolic pathways and further screened the top 100 genes with the most pronounced expression differences within these components and pathways. These genes were primarily associated with GO terms such as response to heat, cellular response to heat, DNA-binding transcription factor activity, response to temperature stimulus, and transcription from RNA polymerase II promoter in response to heat stress. Additionally, they were enriched in KEGG pathways including plant–pathogen interaction, MAPK signaling pathway-plant, plant hormone signal transduction, phenylpropanoid biosynthesis, and photosynthesis-antenna proteins (Figure 7A–D and Tables S8 and S9).

Further Venn diagram analysis of the top 100 genes screened from each group revealed that 16 genes (*PH02Gene26751*, *PH02Gene22223*, *PH02Gene10746*, *PH02Gene25672*, *PH02Gene14139*, *PH02Gene09089*, *PH02Gene32841*, *PH02Gene02139*, *PH02Gene16951*, *PH02Gene03498*, *PH02Gene48116*, *PH02Gene23029*, *PH02Gene09168*, *PH02Gene06266*, *PH02Gene17166*, *PH02Gene10747*) were common to both KEGG and GO analyses, suggesting their potential role as key genes in Moso bamboo’s response to heat stress (Figure 7E and Table S9).

**Figure 8:**
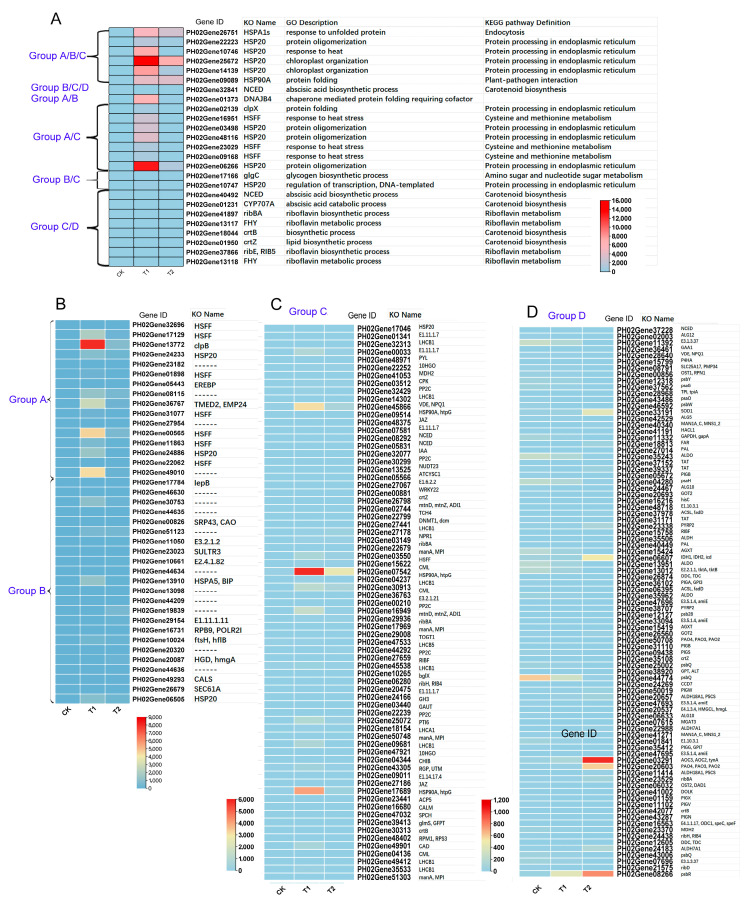
Heatmap analysis of the expression of key genes in key GO and KEGG metabolic pathways between comparative groups of transcriptome data. (**A**) Key differentially expressed genes common to both GO and KEGG enrichment analyses in multiple sample analysis groups. (**B**) Key genes in groups A and B. (**C**) Key differentially expressed genes in group C. (**D**) Key differentially expressed genes in group D. Groups A and B represent the top 100 differentially expressed genes in GO enrichment analysis in sample groups T1 vs. CK and T2 vs. CK, and groups C and D represent the top 100 differentially expressed genes in KEGG enrichment analysis in T1 vs. CK and T2 vs. CK, respectively.

To elucidate the significance and accuracy of the metabolic pathways, cellular components, and key genes identified in Figure 7, we examined the changes in the expression levels of the top 100 genes exhibiting the most pronounced alterations across different subgroups using transcriptome data. As depicted in Figure 8, the 16 genes shared by both GO and KEGG enrichment analyses predominantly belonged to the HSP, HSF, and NCED gene families (Figure 8A). The remaining genes were homologs of EREBP, HSP, CPK, and JAZ genes (Figure 8B–D), which are implicated in biological processes such as cellular response to heat stress, hormone signaling transduction, and secondary metabolite synthesis.

**Figure 9:**
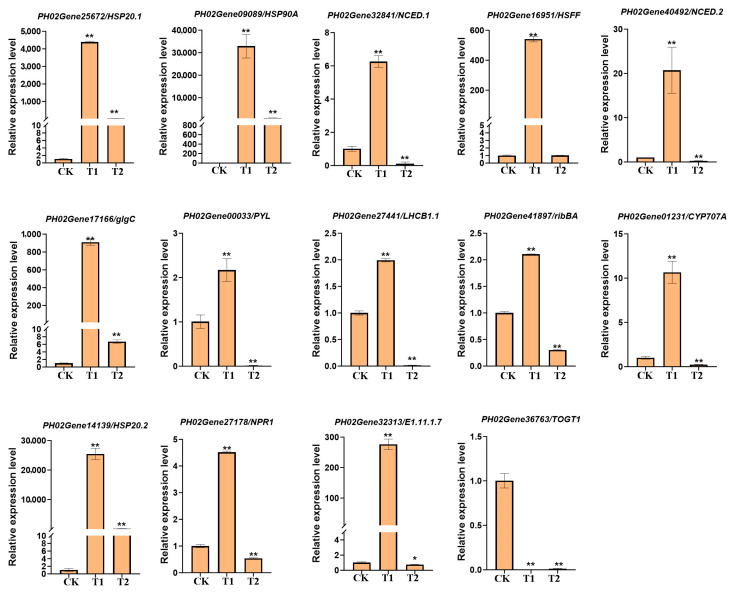
qRT-PCR validation of 14 DEGs involved in the heat stress response in Moso bamboo. Error bars indicate averages of measurements from at least three biological replicates, and asterisks indicate significant differences. Significant differences were analyzed using the one-way ANOVA method, * *p* < 0.05, ** *p* < 0.01.

To validate the expression accuracy of the key genes identified in Figure 8 and RNA-seq analysis, we randomly selected 14 key differentially expressed genes for qRT-PCR validation. These genes, which are affiliated with diverse cellular components and metabolic pathways (as listed in Table S10), include *HSP20 (PH02Gene25672), HSP20 (PH02Gene14139), HSP90A (PH02Gene09089), NCED1.1 (PH02Gene32841), HSFF (PH02Gene16951), glgC (PH02Gene17166), PYL (PH02Gene00033), NPR1 (PH02Gene27178), E1.11.1.7 (PH02Gene32313), bglX (PH02Gene10265), LHCB1 (PH02Gene27441), LHCB1 (PH02Gene32429), TOGT1 (PH02Gene36763), NCED1.2 (PH02Gene40492), CYP707A (PH02Gene01231),* and *ribBA (PH02Gene41897)*. These genes are implicated in metabolic pathways such as protein processing in the endoplasmic reticulum, plant–pathogen interaction, plant hormone signal transduction, phenylpropanoid biosynthesis, carotenoid biosynthesis, and photosynthesis-antenna proteins, all of which are intricately linked to cellular metabolism and responses to adversity stress. The qRT-PCR results further substantiate their significance in the heat stress response process and confirm the accuracy of the RNA-seq analysis of bamboo (Figure 9). This study offers a novel perspective for the cultivation of stress-resistant bamboo varieties.

## 4. Discussion

The transcriptional regulatory networks of plants involved in the response to and recovery from high-temperature stress are complex and precise, involving multiple biological processes and genes [27,28]. Through a transcriptome sequencing analysis, multiple genes have been identified in various species, such as potato [29] and switchgrass [30,31]. The heat stress response of Moso bamboo, an economically and ecologically important bamboo species, has also received considerable research attention. However, the molecular mechanism of its response to high temperatures needs to be further studied. In this study, Moso bamboo was exposed to high-temperature stress for varying durations, and transcriptome analysis was conducted to identify key genes involved in the heat stress response. The findings provide valuable insights into the genetic and molecular mechanisms underlying heat tolerance in Moso bamboo.

This study systematically examined the indicators related to the photosynthetic rate in Moso bamboo after heat stress treatment, and the results clearly showed that the high-temperature environment had a significant inhibitory effect on its photosynthesis (Figure 1). Through the functional annotation of single genes using GO and KEGG, the diversity and complexity of cellular components, molecular functions, and biological processes underlying stress responses were revealed. In the leaves of Moso bamboo, significant changes in DEGs were observed between the control group (CK) and the recovery group under heat stress treatment, suggesting the existence of a complex regulatory network responsible for coordinating the plant’s response mechanism to heat stress.

Heat shock proteins (HSPs), a class of important molecular chaperones highly conserved in the plant kingdom, play a crucial role in maintaining proper protein folding, stability, and cellular homeostasis under heat stress [32,33,34]. The identified HSPs cover multiple families across various species, such as *Arabidopsis thaliana*, with 21 genes encoding heat shock transcription factors (HSFs) [35], and wheat, with as many as 56 [18]. These HSPs play a crucial role in maintaining cellular homeostasis under prolonged heat exposure by performing diverse biological functions that prevent cellular toxic damage. For example, overexpression of HSFs in *Arabidopsis* has been shown to significantly enhance plant tolerance to heat and other stresses, and HsfA3 overexpression in *Arabidopsis* has been demonstrated to increase thermotolerance by regulating the expression of heat-inducible genes and antioxidant enzymes [36,37]. Additionally, 25 *ZmHsf* and 22 *ZmHsp70* genes have been identified, which may potentially influence heat stress responses [38]. In this study, multiple HSPs and HSFs, such as *PH02Gene25672/HSP20.1, PH02Gene09089/HSP90A, PH02Gene16951/HSFF*, and *PH02Gene14139/HSP20.2*, showed significant upregulation trends after 1 h and 24 h of heat stress treatment, comprehensively demonstrating their pivotal role in safeguarding cellular proteins via chaperone activity and mirroring the plant’s immediate adaptive responses to the detrimental effects of heat stress on cellular components, such as membrane fluidity regulation and protein homeostasis maintenance (Figure 8 and Figure 9). We identified the metabolic pathways and cellular components with the most significant differentially expressed genes after GO and KEGG enrichment analysis in Moso bamboo under heat stress treatment.

We further selected the top 10 metabolic pathways and 100 genes with the most significant expression changes in the cellular components from each respective group (Figure 7 and Figure 8). A small number of these genes were shared among the groups, while the majority were unique to their respective groups, indicating the stage-specific responses and complex regulatory relationships involved in the response to heat stress signals in Moso bamboo. In addition to the most commonly studied genes, such as HSPs and HSFs, we identified numerous genes from other metabolic pathways, for example, *PH02Gene32841/NCED.1* and *PH02Gene40492/NCED.2,* which are involved in the abscisic acid (ABA) biosynthesis pathway and the carotenoid metabolic pathway, which participate in ABA-mediated heat stress responses. *E1.11.1.7/PH02Gene32313* is involved in the phenylpropanoid metabolic pathway. *PH02Gene27441/LHCB1.1* belongs to the photosynthesis-antenna protein pathway, participating in the photosynthetic response under heat stress (Figure 8 and Figure 9). These findings offer valuable insights that can be leveraged to enhance plant responses to heat stress. In future genetic breeding studies, researchers may consider overexpressing or knocking out these genes to regulate cellular osmotic pressure, promote the synthesis of secondary metabolites, and strengthen cell walls-ultimately improving plant stress resistance.

## 5. Conclusions

In this study, we integrated transcriptome sequencing, differential gene expression analysis, and functional enrichment to systematically screen for genes that play a key role in response to heat stress in Moso bamboo. Through comparing leaf samples stressed at 42 °C for 0 h (control), 1 h (acute stress), and 24 h (continuous stress), we revealed temporal changes in gene regulation, including the activation of heat stress proteins (HSPs) and transcription factors (e.g., HSF, MYB, and NAC families), as well as stress-associated pathways, such as the photosynthetic pigment synthesis, MAPK signaling, and phenylpropane biosynthesis pathways. The key candidate genes, such as *HSP20 (PH02Gene25672), HSP20 (PH02Gene14139), HSP90A (PH02Gene09089)*, *NCED1.1 (PH02Gene32841)* and so on, were further validated using qRT-PCR to confirm their expression changes in response to heat stress. This study not only deepens the understanding of the molecular mechanism of heat stress in Moso bamboo but also provides targets for breeding stress-resistant varieties to help achieve carbon neutrality in the context of global warming.

## Data Availability

The original contributions presented in this study are included in the article/Supplementary Materials. Further inquiries can be directed to the corresponding authors.

## Supplementary Materials

The following supporting information can be downloaded at https://www.mdpi.com/article/10.3390/genes16080855/s1, Table S1. The primer used in this study; Table S2. Sequencing data statistics; Table S3. Statistical table of transcriptome sequencing data comparison results; Table S4. Functional annotation statistics table; Table S5. Tactor identification statistics; Table S6. Expression differential expression gene statistics; Table S7. Transcriptome sequencing matrix; Table S8. GO and KEGG gene set enrichment chordal maps for Figure 7; Table S9. Venn analysis of gene sets after GO and KEGG enrichment analysis in T1 vs. CK and T2 vs. CK groups for Figure 7; Table S10. qRT-PCR validation of gene selection.
